# Effectiveness of Mindfulness-Based Cognitive Therapy in reducing psychological distress and improving sleep in patients with Inflammatory Bowel Disease: study protocol for a multicentre randomised controlled trial (MindIBD).

**DOI:** 10.1186/s40359-023-01127-0

**Published:** 2023-06-19

**Authors:** Milou M ter Avest, Annelieke S M van Velthoven, Anne E M Speckens, Gerard Dijkstra, Martin Dresler, Carmen S Horjus, Tessa E H Römkens, Ellen M Witteman, Willemijn A van Dop, Quirine M Bredero, Loes H C Nissen, Marloes J Huijbers

**Affiliations:** 1 Department of Psychiatry, Centre for Mindfulness, Radboud University Medical Centre, Postbus 9101, Nijmegen, 6500 HB the Netherlands; 2 Donders Centre for Medical Neuroscience, Donders institute for Brain, Cognition and Behaviour, Radboud University Nijmegen, Nijmegen, the Netherlands; 3 Department of Gastroenterology and Hepatology, Jeroen Bosch Hospital, ‘s-Hertogenbosch, the Netherlands; 4 Department of Gastroenterology and Hepatology, University Medical Centre Groningen, University of Groningen, Groningen, the Netherlands; 5 Department of Gastroenterology and Hepatology, Rijnstate Hospital, Arnhem, the Netherlands; 6 Department of Gastroenterology and Hepatology, Canisius Wilhelmina Hospital, Nijmegen, the Netherlands; 7 Department of Gastroenterology and Hepatology, Radboud University Medical Centre, Nijmegen, the Netherlands; 8 Department of Health Psychology, University Medical Centre Groningen, University of Groningen, Groningen, the Netherlands

**Keywords:** Mindfulness, Mindfulness-Based Cognitive Therapy, Inflammatory Bowel Disease, Crohn’s disease, Ulcerative colitis, Randomised controlled trial, Study protocol, Psychological distress, Sleep quality, (Cost-)effectiveness

## Abstract

**Background:**

Many patients with Inflammatory Bowel Diseases (IBD) suffer from psychological distress, fatigue and sleep disturbances, which are associated with reduced quality of life (QoL) and increased societal costs. Only limited psychosocial treatment options are available. As Mindfulness-Based Cognitive Therapy (MBCT) has demonstrated to improve psychological distress, QoL and sleep in other populations, MBCT might also be effective in patients with IBD.

**Methods:**

The MindIBD study is a prospective, multicentre, randomised controlled trial comparing MBCT plus Treatment As Usual (TAU) versus TAU alone in a targeted number of 136 IBD patients in remission, aged 16 years and older with at least mild psychological distress (Hospital Anxiety and Depression Scale (HADS) total score ≥ 11). Primary outcome is reduction of psychological distress post-intervention, measured by the HADS. In addition, the effect of MBCT on sleep quality (including actigraphy and electroencephalography recordings), fatigue, disease activity, perceived disease control, QoL and positive mental health will be examined. Assessments will be conducted at baseline and at 3, 6, 9 and 12 months follow-up. Cost-effectiveness will be determined and a process evaluation will be conducted.

**Discussion:**

This study will provide valuable insight into the clinical effect of MBCT on psychological distress, sleep quality, fatigue and QoL in IBD patients and into the cost-effectiveness. If effective, MBCT can be a valuable addition to the available psychosocial interventions for patients with IBD. Moreover, findings from this study may also be applicable in patients with other chronic conditions.

**Trial registration:**

ClinicalTrials.gov: NCT04646785, registered on 30/11/2020.

**Supplementary Information:**

The online version contains supplementary material available at 10.1186/s40359-023-01127-0.

## Background

Inflammatory Bowel Disease (IBD), mainly consisting of Crohn’s disease and ulcerative colitis, is characterised by chronic relapsing inflammation of the intestinal tract. In the Netherlands, currently about 90,000 patients have been diagnosed with IBD and the prevalence seems to be rising worldwide [1]. The disease manifests mainly in young adults, aged between 15 and 30 years, who are in a turbulent part of their life, finding their way with housing, friends, education and perhaps starting a family and career. Physical symptoms include abdominal pain, diarrhoea, rectal bleeding and fatigue. Although the arsenal of drugs has increased in past decades, there is no cure for the disease and patients are usually treated with different (immunosuppressive) medications for a long time. Despite these treatment options, flares are common with cumulative rates of 69–93% after 5 years for patients with Crohn’s disease and 78–82% for patients with ulcerative colitis [2].

Besides physical symptoms, many IBD patients experience psychological distress, including symptoms of depression and anxiety. This could be due to its unpredictable course and prognosis, difficulties accepting their chronic disease and side effects of medication [3]. About a quarter of patients in remission experience depressive symptoms and about a third experience symptoms of anxiety, which is two to three times higher than healthy controls [4]. These numbers nearly double during flares [3, 4]. Psychological distress has been associated with a decrease in self-care and quality of life (QoL) and an increase in the experience of physical symptoms, including fatigue. Besides that, these patients are more likely to seek medical care, which leads to higher healthcare costs [3]. Moreover, the association between psychological distress and disease activity might be bidirectional, since it has been suggested that perceived psychological distress may affect the onset and course of IBD [5]. However evidence is scarce and inconsistent [5, 6].

In addition, many IBD patients suffer from fatigue, sleep disturbances and decreased QoL. In quiescent IBD, half of the patients experience fatigue, compared to 8% in a healthy population [7]. Across several countries, and in particular from the patient’s perspective, fatigue has been recognised as a key topic for research [8]. Moreover, patients with IBD report poorer sleep quality than healthy controls [9]. Sleep disturbances have been linked to disease activity and shown as a marker for subclinical inflammation and a risk factor for developing a flare [10]. It is therefore not surprising that IBD patients report lower QoL compared to the general population and this underscores the importance of finding appropriate treatment options that address these symptoms [11].

Mindfulness-based interventions (MBIs) have been shown to be effective in reducing psychological distress and fatigue, as well as improving sleep quality and QoL, both in patients with mental and somatic illnesses [12, 13]. MBIs are psychosocial group-based interventions that focus on the cultivation of mindfulness skills in order to better cope with negative, repetitive thoughts and feelings [14]. The effect of MBIs on psychological distress is potentially mediated by mechanisms such as mindfulness, self-compassion, rumination and worry [15]. Sleep quality might also play a role, given the association between poor sleep quality and psychological distress [16], and the positive effect mindfulness appears to have on sleep quality [13].

Although the available evidence on the effectiveness of MBIs for IBD is still relatively scarce, a meta-analysis, which included 477 patients in eight randomised controlled trials (RCTs), showed significant benefits in terms of psychological distress and QoL [17]. However, the variety in MBIs was rather high, none of the studies had psychological distress as primary outcome, and most studies suffered from methodological limitations such as small sample size, incomplete data reporting and a lack of fidelity measures. Looking specifically to the available RCTs using Mindfulness-Based Cognitive Therapy (MBCT) or Mindfulness-Based Stress Reduction (MBSR) in IBD patients, it was limited to two studies [18, 19]. Jedel et al. included patients with a recent flare of ulcerative colitis and found no difference in disease course between the MBSR and control groups, although patients in the intervention group experienced less stress during a flare in the follow-up period than those in the control group [18]. Schoultz et al. found a significant decrease in psychological distress and non-significant improvement of QoL and disease activity in patients with IBD allocated to MBCT compared to the waitlist control condition [19]. Two additional RCTs examining MBCT in IBD were conducted after the meta-analysis [20, 21]. Ewais et al. showed that depressed young adults with IBD were less depressed and stressed after MBCT, with an improvement in coping but not in QoL [20]. Finally, in a recent study of Bredero et al., fatigued IBD patients experienced significantly lower levels of fatigue after MBCT compared to the waitlist group [21]. In none of these studies sleep quality was an outcome of interest. Due to the limited evidence, it seems warranted to conduct further research to the effectiveness of mindfulness in patients with IBD, particularly focused on psychological distress and sleep quality.

The MindIBD study is a multi-centre RCT, using a variety of subjective and objective outcome measures. The trial primarily focuses on the effectiveness of MBCT in reducing psychological distress in patients with IBD. Besides that, we are also interested in the effects on sleep quality, fatigue, disease activity, perceived disease control, QoL and positive mental health. In addition, we will investigate the possibly mediating role of mindfulness, self-compassion and perseverative thinking. Eventually, cost-effectiveness will be examined and a process evaluation of implementing MBCT in IBD healthcare will be conducted.

## Methods

### Study design

The MindIBD trial is a prospective, multicentre, parallel-group RCT comparing MBCT plus treatment as usual (TAU) versus TAU alone. Assessments are conducted at baseline (T0) and at 3 (T1), 6 (T2), 9 (T3) and 12 (T4) months follow-up. To evaluate the feasibility for implementing MBCT in the care for patients with IBD, a process evaluation with qualitative interviews will be part of the project. The study protocol has been approved by the ethical review board CMO Arnhem-Nijmegen (#2021–7319; NL75762.091.20).

### Setting

Participants are recruited from the gastroenterology and paediatrics outpatient clinics of four Dutch hospitals, which consist of one university hospital (Radboud University Medical Centre, Nijmegen) and three general teaching hospitals (Jeroen Bosch Hospital (‘s-Hertogenbosch), Canisius Wilhelmina Hospital (Nijmegen) and Rijnstate Hospital (Arnhem)).

### Study population

The study population consists of patients of 16 years and older with a confirmed IBD diagnosis of Crohn’s disease, ulcerative colitis or indeterminate colitis. Additional inclusion criteria are: (1) IBD in remission (faecal calprotectin < 250 mg/kg [22]) for at least three months; (2) taking no IBD medication or being on a stable dose of aminosalicylates, immunosuppressants, biologics or small molecule therapy for at least three months prior to enrolment; (3) a Hospital Anxiety and Depression Scale (HADS) total score ≥ 11, which has been previously used to select somatically ill patients with elevated levels of anxiety or depression [23, 24]; and (4) a good understanding of the Dutch language. The following exclusion criteria are applied: (1) severe psychiatric disorders, e.g. acute suicidality, psychosis; (2) current alcohol or drug dependency; (3) anaemia (haemoglobin level range in men: 8.4–11.0 mmol/L and in women: 7.4–10.0 mmol/L); and (4) prior participation in an eight-week MBI.

### Procedure and assessments

**Fig. 1:**
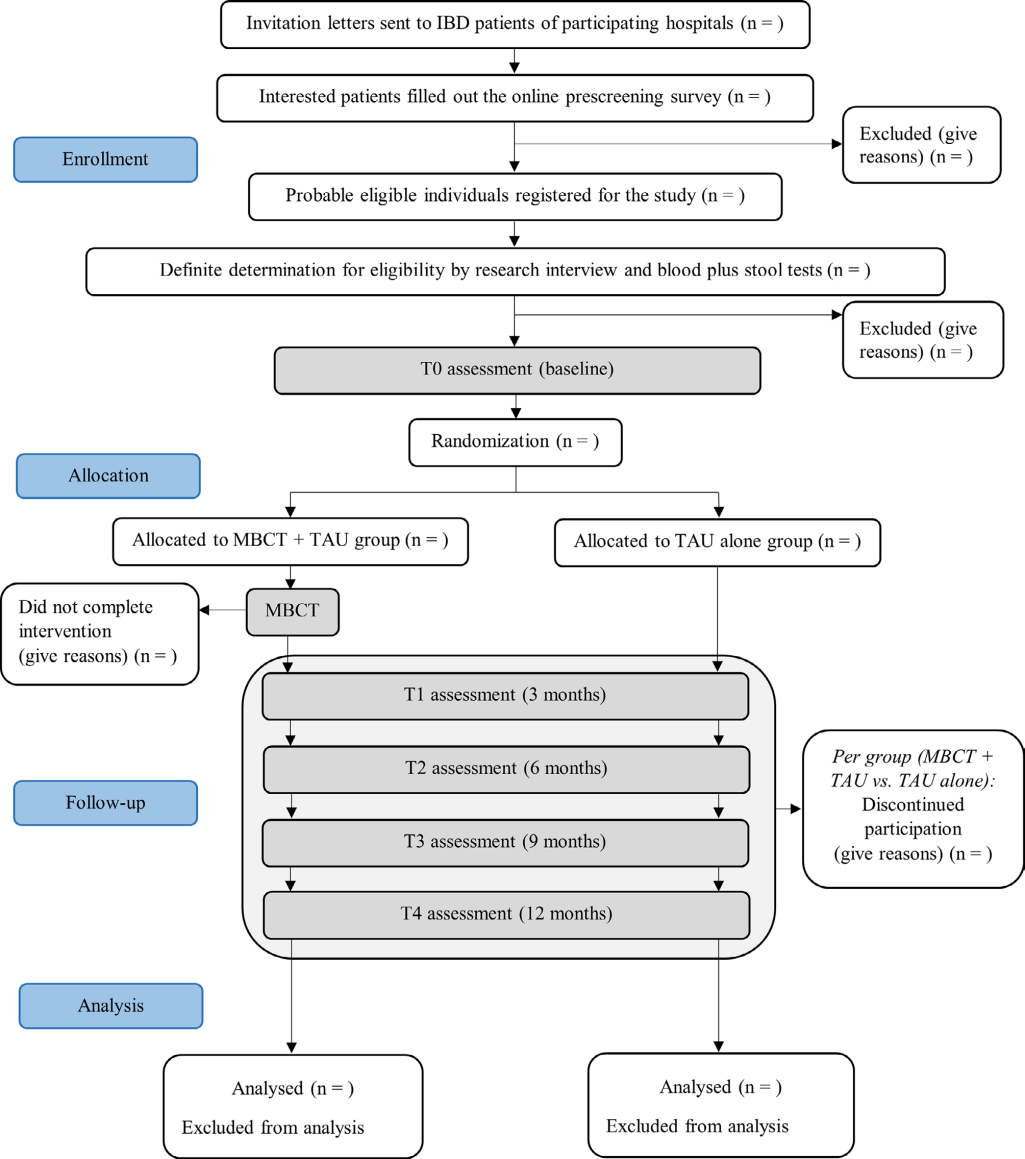
Flowchart of study procedure, based on CONSORT diagram

Figure 1 provides a flowchart of the study procedure. In order to minimize the risk of selection bias by attending physicians, all IBD patients treated in these hospitals receive an invitation leaflet. Those interested in participation are referred to an online, anonymous pre-screening survey [25], which includes the HADS and questions about the main eligibility criteria. Probably eligible participants are guided to a registration form, after which they receive additional information about the trial and are invited for a research interview. During this research interview the study is explained in more detail, the inclusion and exclusion criteria are reviewed thoroughly and written informed consent is obtained (Appendix 1). Definite eligibility is determined after laboratory screening to rule out anaemia or elevated faecal calprotectin levels. After definite inclusion in the study, participants are invited for the baseline assessment, which consists of: (1) self-report questionnaires; (2) an interview in which demographic and disease characteristics are examined (age, sex, country of origin, level of education, marital status, employment status, body mass index, IBD type, medication use, age at onset of IBD, time since last flare, hospitalizations due to flare, comorbid psychiatric diagnoses); and (3) sleep analysis, using sleep electroencephalography (EEG) headband for three consecutive nights. From baseline until post-treatment, participants are asked to wear an actigraphy wristband for additional sleep data. At post-treatment, they are asked to sleep another three nights with the EEG headband. The post-treatment assessment (3 months after baseline) and the follow-up assessments (6, 9 and 12 months after baseline) consist of self-report questionnaires and an interview by clinician. The 6- and 12-month assessments include blood and stool samples for all participants. The 3- and 9-month assessment includes blood and stool samples only for patients with a suspected flare. Table 1 provides an overview of assessments and measures. The content of the outcome measures is explained below under the heading ‘outcome measures’.

**Table 1:** Overview of assessments and measures

	T0 (baseline)	MBCT	T1 (3 months)	T2 (6 months)	T3 (9 months)	T4 (12 months)
**Self-report questionnaires**						
*Psychological distress*						
HADS	✓		✓	✓	✓	✓
*Sleep quality and fatigue*						
PSQI	✓		✓	✓	✓	✓
ISI	✓		✓	✓	✓	✓
FACIT-F	✓		✓	✓	✓	✓
*IBD-related measures*						
SIBDQ	✓		✓	✓	✓	✓
IBD-control	✓		✓	✓	✓	✓
*Mental health-related measures*						
PTQ	✓		✓	✓	✓	✓
FFMQ-SF	✓		✓	✓	✓	✓
SCS-SF	✓		✓	✓	✓	✓
MHC-SF	✓		✓	✓	✓	✓
MAQ		✓	✓	✓	✓	✓
*Cost-effectiveness*						
TiC-P	✓		✓	✓	✓	✓
EQ-5D	✓		✓	✓	✓	✓
**Clinical interview**						
HBI/SCCAI	✓		✓	✓	✓	✓
Time to flare	✓		✓	✓	✓	✓
(S)AE	✓		✓	✓	✓	✓
**Clinical outcomes**						
Blood tests^*^	✓		(✓)	✓	(✓)	✓
Faecal calprotectin	✓		(✓)	✓	(✓)	✓
**Sleep analysis**						
Headband (*Zmax*)	✓		✓			
Wristband (*Fitbit*)	✓	✓	✓			

*Abbreviations*: EQ-5D, EuroQol-5D-5 L; FACIT-F, Functional Assessment of Chronic Illness Therapy-Fatigue; FFMQ-SF, Five Facet Mindfulness Questionnaire-Short Form; HADS, Hospital Anxiety and Depression Scale; HBI, Harvey Brashaw Index; IBD, Inflammatory Bowel Disease; ISI, Insomnia Severity Index; MAQ, Mindfulness Adherence Questionnaire; MBCT, Mindfulness-Based Cognitive Therapy; MHC-SF, Mental Health Continuum-Short Form; PSQI, Pittsburgh Sleep Quality Inventory; PTQ, Perseverative Thinking Questionnaire; (S)AE, (Serious) Adverse Events; SCCAI, Simple Clinical Colitis Activity Index; SCS-SF, Self-Compassion Scale-Short Form; SIBDQ, Short Inflammatory Bowel Disease Questionnaire; TiC-P, Trimbos iMTA Questionnaire for Costs associated with Psychiatric Illness

*Blood tests consists of haemoglobin, C-reactive protein and albumin. At baseline ferritin is added.

### Randomisation and blinding

Randomisation is computerised using CastorEDC [26], an Electronic Data Capture program complying with all regulatory guidance [27]. Participants are allocated using a 1:1 randomisation ratio scheme. Randomisation is stratified for hospital, sex and type of IBD. In order to ensure balanced groups, block randomisation with block sizes of either 2, 4 or 6 are used. Participants cannot be blinded to allocation. For reasons of coordination and logistics, randomisation is performed by a member of the research team, and the research team has therefore access to the information about the assigned group.

### Intervention: MBCT

The MBCT program is based on the protocol published by Segal, Williams and Teasdale [14]. The program consists of eight weekly 2.5 hour sessions, a six hour silent day between session 6 and 7, and daily home practice of 45 minutes guided by audio files. Groups consist of eight to ten participants. Materials are adjusted to include IBD-related examples. The course is offered in the cities where patients are treated (Nijmegen, Arnhem and ‘s-Hertogenbosch) and taught by qualified teachers according to the advanced criteria of the Association of Mindfulness-Based Teachers in the Netherlands and Flanders [28]. These criteria include a minimum of 150 hours of education in MBSR/MBCT (entailing theoretical background, skills practice, supervision and reflection), a minimum of three years of personal meditation practice and attending retreats, and providing a minimum of two courses per two years. All mindfulness teachers participated in a kick-off meeting before the start of the trial, in which the trial-specific MBCT protocol was discussed. Peer supervision meetings are organised twice per MBCT course during the intervention phase of the trial. Teaching sessions are recorded and a random sample of sessions will be rated by two independent raters using the Mindfulness-Based Interventions: Teaching Assessment Criteria (MBI:TAC) [29]. Participation of the training can be discontinued on participant’s request at any time. If so, the participant is encouraged to complete further assessments of the study.

### Comparator: treatment as usual

TAU consists of all healthcare that patients usually receive to treat IBD according to Dutch and European treatment guidelines [30–33]. These treatments mainly focus on pharmacological and surgical disease control and prevention of complications. TAU can also include non-mindfulness based psychosocial treatments. All healthcare use is registered (see outcome measures, cost-effectiveness). After the final assessment, participants of the TAU group will also be given the opportunity to participate in an MBCT course.

### Outcome measures

#### Primary outcome

#### HADS

The primary outcome is psychological distress at post-treatment, measured by a total score of the *HADS*, which was originally developed as a screening instrument for the possible anxiety and depressive states in medical out-patient clinics [34]. The questionnaire consists of 14 items of which 7 items are related to depression and 7 items are related to anxiety. Scores for each item ranges from 0 to 3, giving total scores between 0 and 42 (and maxima of 21 per subscale). Higher scores indicate higher levels of psychological distress. The internal consistency of the HADS-total in the Dutch version of the questionnaire has been shown to be good (Cronbach’s *α* = 0.82–0.90) [35].

#### Secondary outcomes

#### Sleep quality and fatigue

The *Pittsburgh Sleep Quality Inventory (PSQI)* is used to measure subjective sleep quality [36]. The PSQI consist of 19 items offering 7 component scores (subjective sleep quality, sleep latency, sleep duration, habitual sleep efficiency, sleep disturbances, use of sleeping medication and daytime dysfunction) and a composite score. Scores for each component range from 0 to 3 [36]. The overall PSQI score is then calculated by summing up the 7 component scores, providing an global score ranging from 0 to 21. Higher scores indicate worse sleep quality. Cronbach’s *α* has been shown to be acceptable (0.64–0.83) in different samples, including chronic diseases such as rheumatic arthritis (Cronbach’s *α =* 0.73) [37].

The 7-item *Insomnia Severity Index (ISI)* is used to measure insomnia, including questions about the nature, severity and impact [38]. A 5-point Likert scale is used to score each item, providing a total score ranging from 0 to 28. Higher scores reflect greater insomnia severity. The ISI has been shown to have a good reliability in quantifying perceived insomnia severity (Cronbach’s *α* = 0.70–0.92) [39].

Objective sleep quality is measured electrophysiologically using a 2-channel EEG headband (*Zmax, Hypnodyne*) and by indirect parameters using an actigraphy wristband (*Fitbit Activity Tracker, Google*). From the sleep EEG data, classical sleep scoring parameters [40] related to sleep quality can be derived: total sleep time (TST), time in bed (TIB: period from lights out/start recording to lights on/stop recording in minutes), sleep efficiency (SE: defined as the percentage of TST/TIB*100%), sleep onset latency (SOL: from lights out/start recording to first epoch of any sleep stage in minutes), wake after sleep onset (WASO: the number of minutes awake following the first three consecutive epochs of any sleep stage), absolute and relative duration of rapid eye movement sleep (REM sleep), and absolute and relative duration of deep sleep. Sleep stage analysis per 30 seconds epoch plus derived variables of the *Zmax* sleep EEG recordings will be performed semi-automatically using in-house software [41]. The following sleep scoring parameters can be derived from the *Fitbit *sleep data: TST, WASO, absolute and relative duration of REM sleep, and absolute and relative duration of deep sleep. Although polysomnography is the gold standard of measuring sleep, comparable two-channels sleep EEG headbands demonstrate good validity [42], and actigraphy via fitness trackers has been shown valid for screening purposes if applied repeatedly [43]. Currently, an in-house validation study of the *Zmax* is carried out.

The *Functional Assessment of Chronic Illness Therapy-Fatigue (FACIT-F)* is a measure to assess subjective fatigue and its impact on daily life [44]. The questionnaire consists of 40 items, divided into 13 items about fatigue and 27 items about four generic domains of health-related QoL (physical well-being, social/family well-being, emotional well-being and functional well-being) [45]. All items are scored on a 5-point Likert scale. Higher scores in the fatigue subscale (range: 0–52) indicate less fatigue. The four generic domains together reflect better quality of life, ranging from 0 to 108. The subscale fatigue can be used as a stand-alone questionnaire to assess subjective fatigue (Cronbach’s *α* = 0.93–0.95) [44], and has been shown excellent reliability in patients with IBD (Cronbach’s *α* = 0.94) [46]. The QoL-questions has been shown to be reliable in chronic diseases, such as multiple sclerosis (Cronbach’s *α* = 0.82–0.96) [47].

#### IBD-related measures

The *Short Inflammatory Bowel Disease Questionnaire (SIBDQ)* is used to measure IBD-related QoL [48]. The SIBDQ has 10 items scored on a 7-point Likert scale, grouped into bowel symptoms, systemic symptoms, emotional function and social function. Overall scores range from 10 to 70, with higher scores reflecting better QoL. The SIBQ has been shown to be reliable (Cronbach’s *α* = 0.78) [49].

The *IBD-Control* is used to measure disease control from the patient’s perspective [50]. The IBD-Control questionnaire consists of 13 items and a visual analogue scale, ranging 0-100 (0 = worst control). The 13 items have 3 answer options and include questions about energy, sleep, mood, freedom of discomfort, ability to perform daily activities and treatment concerns. Overall scores ranges from 0 to 26, where lower scores indicates poorer control. The psychometric properties of the IBD-control have been shown to be good (Cronbach’s *α* = 0.85) [50], including the shortened IBD-control-8 version in a Dutch population (Cronbach’s *α* = 0.82) [51].

Clinical disease activity is assessed by clinician-based questionnaires: *Harvey-Bradshaw Index* (HBI) for Crohn’s disease and *Simple Clinical Colitis Activity Index* (SCCAI) for ulcerative colitis [52, 53]. HBI consist of 12 questions about general well-being (score 0–4), abdominal pain (score 0–3), soft stools (1 point per soft stool), abdominal mass (score 0–3) and extra-intestinal manifestations (score 0–1 per manifestation, 8 in total). A flare may be suspected at a score of 5 or higher. SSCAI consist of 9 questions about general well-being (score 0–4), bowel frequency (score 0–2), urgency (score 0–3), rectal blood loss (score 0–3) and extra-intestinal manifestations (score 0–1 per manifestation, 4 in total). In case of a score of 3 or higher, it may indicate a flare.

*Time to flare* is assessed based on the clinical interviews. If a participant reports that they have (had) a flare, the medical record will be tracked. The assessment of the attending physician is leading. A flare is defined by elevated faecal calprotectin levels (> 250 mcg/g) which is validated by a clinical interview plus commencement of any new IBD medication or altered dosing of existing IBD medication. We consider the date of elevated faecal calprotectin levels or change in IBD medication as the start of the flare.

Laboratory tests will be performed every 6 months (T0, T2 and T4), following the regular medical check-ups [54] and extra in case a flare is suspected (HBI ≥ 5, SCCAI ≥ 3). These tests consist of faecal calprotectin, haemoglobin, C-reactive protein and albumin. Having higher levels of faecal calprotectin and C-reactive protein plus low albumin may indicate disease activity [54–56].

#### Mental health-related measures

The *Perseverative Thinking Questionnaire* (PTQ) is used to measure repetitive negative thinking [57]. The PTQ is a 15-item self-report questionnaire, comprising statements about 3 processes: core features (repetitive, intrusive and difficult to disengage thoughts), unproductiveness and capturing mental capacity. Each item is rated on a 5-point Likert scale, resulting in a global repetitive negative thinking score ranging from 0 to 60. Higher scores indicate more perseverative thinking. The Dutch version of the questionnaire has been shown to have excellent reliability (Cronbach’s *α* = 0.94) [58].

The *Five Facet Mindfulness Questionnaire-Short Form* (FFMQ-SF) is used to measure mindfulness skills [59]. The FFMQ-SF consists of 24 items divided into 5 facets: observing, describing, acting with awareness, non-judging and non-reactivity. Each item has 5 answer options, and higher scores indicate more mindfulness skills. All facets of the Dutch version in a sample with depressive symptomatology has been shown to be reliable (Cronbach’s *α* = 0.73–0.91) [60].

The 12-item *Self-Compassion Scale–Short Form* (SCS–SF) consist of 6 scales to measure self-compassion: self-kindness, self-judgment, common humanity, isolation, mindfulness and over-identification [61]. Each scale consists of 2 items, using a 7-point Likert scale. A total self-compassion score is computed by reversing the negative subscale items (self-judgment, isolation, over-identification) and then combining the subscale scores. Higher scores indicate more self-compassion. The psychometric properties of the Dutch SCS-SF have been shown to be good (Cronbach’s *α* = 0.87) [61].

The *Mental Health Continuum-Short Form* (MHC-SF) is a 14-item self-report questionnaire that measures 3 dimensions of positive mental health: emotional well-being, psychological well-being and social well-being [62]. Items are scored on a 6-point Likert scale. All items are summed and higher scores indicate greater levels of positive mental health. The internal consistency of the total score of the Dutch version of this questionnaire has been shown to be very good (Cronbach’s *α* = 0.89) [63].

The *Mindfulness Adherence Questionnaire* (MAQ) is a 12-item questionnaire about mindfulness practice [64]. The first 2 items measure formal practice in terms of frequency and average duration of practice. The remaining items measure the quality of formal practice. Items are scored on a 7-point Likert-scale. Higher subscale scores reflect higher practice quality for that respective practice type. Because a Dutch version of the questionnaire was not available yet, we translated and back-translated the original MAQ for the purpose of this study. The reliability of the English version has been shown to be adequate (Cronbach’s *α* = 0.79) [64].

#### Cost-effectiveness

Cost-effectiveness is investigated using the *Trimbos and iMTA questionnaire for Costs associated with Psychiatric illness* (TiC-P) as a reliable and valid measure of healthcare utilization and productivity losses at work and in daily life [65, 66]. Unit cost estimates will be derived from the national manual for cost prices in the healthcare sector [67].

Health-related QoL, using the *EuroQol-5D-5L* (EQ-5D), is used to assess quality adjusted life years [68]. The EQ-5D consist of 5 dimensions: mobility, self-care, usual activities, pain/discomfort and anxiety/depression. Each dimension has 5 levels: no problems, slight problems, moderate problems, severe problems and extreme problems. The EQ-5D index is calculated by applying predetermined weights to the 5 dimensions, and gives a societal-based global ‘utility score’ of the participant’s health status on a scale between − 0.33 (worse than death) and 1 (perfect health). Based on these utility scores at T0 and T1, Quality-Adjusted Life Years (QALYs) were calculated for each participant with the Area Under the Curve method: ((EQ-5D T0 + EQ-5D T1)/2)*(3/12) and using the Dutch index tariff [69]. This questionnaire has been validated in IBD patients [70].

#### Qualitative interviews

In addition to the questionnaires, a qualitative study is embedded in the project, including a subsample of the trial participants and other stakeholders (e.g. gastroenterologists, mindfulness teachers, health insurance companies, healthcare managers) for in-depth interviews with regard to (a) the usefulness of MBCT in coping with their illness and improving self-care, and (b) the feasibility of MBCT, including possible barriers and facilitators for implementation. Trial participants will be purposively sampled (e.g. disease type, age, sex, compliance with MBCT), including as much diversity in perspectives as possible. We will continue to invite participants until saturation of the data has been reached, i.e. when no new themes are coming up any longer.

### Data management and monitoring

Data will be collected using CastorEDC [26]. Unique participant codes will be assigned to ensure confidentiality. Data that lead to contact details of the participants will be stored in a separate dataset from all other research data collected during the study. Access to the code that couples the study data to the contact details is limited to the principal investigator and a restricted number of researchers. All data will be stored using encrypted digital files and password protected folders with access to a limited number of researchers. The data will be securely stored according to the Personal Data Protection Act for a minimum period of 15 years after the study is completed. During the trial, access to the data will be limited to the researchers. Once trial results are reported, anonymised data will be made available upon reasonable request.

With regard to NFU-classification, the risk of harm in this study is negligible. Therefore a data monitoring committee has been deemed unnecessary for this study. For the same reason, no interim analysis is planned.

### Safety monitoring

In accordance with Good Clinical Practice guidelines, (serious) adverse events ((S)AEs) are reported. Adverse events that may occur are examined at each assessment by explicitly asking participants if they have experienced any adverse events. Although the occurrence of temporary difficulties is not uncommon [71], serious or long lasting effects are expected to be minimal [72]. IBD flares are not classified as adverse events, because these are expected in this specific population [2].

### Statistical analysis

#### Sample size calculation

Based on the most recent meta-analysis of MBCT in IBD, we anticipate the effect size of the reduction of psychological distress to be Cohen’s *d* = 0.51 [17]. With an error probability of 0.05, 0.80 power and a 1:1 allocation ratio, the required total sample size is 98. To account for dropout, we looked at dropout rates in the two similar RCTs of MBIs in patients with IBD, which were 11.5% [18] and 44% [19], respectively. These rates were averaged, i.e. 28%. Using the formula N = n/(1-(z/100)), our corrected sample size is N = 136 (n = 68 per condition).

#### Treatment effects at post-treatment: primary outcome

All analyses will be performed and reported according to the CONSORT guidelines, i.e. primarily on intention-to-treat basis. Treatment effects on the primary outcome (psychological distress at post-treatment, assessed with the HADS) will be analysed using Linear Mixed Effect models, with time, group (MBCT + TAU versus TAU) and Group*Time interaction effects added as fixed effects, while controlling for baseline HADS score and stratification variables (i.e. centre, sex, IBD type). To account for possible dependency caused by clustering in intervention groups, we will add ‘group’ as a random effect if the intra-cluster correlation ≥ 0.05. Restricted maximum likelihood will be used as estimation method to handle missing data. Effect sizes will be calculated using Cohen’s *d*, with values of 0.20–0.50 indicating small effects, 0.50–0.80 moderate effects, and > 0.80 large effects [73]. Cohen’s *d* will be calculated by dividing the adjusted group difference between the means at T1 by the pooled standard deviation at T0.

We will also perform a per-protocol analysis selecting all participants who completed at least half of the MBCT sessions (4 or more) for those assigned to the MBCT condition and inversely, all who have not participated in any mindfulness intervention for those assigned to the TAU condition.

#### Treatment effects at post-treatment: secondary outcomes

Treatment effects on the secondary outcomes (subjective sleep quality (PSQI, ISI), fatigue (FACIT-F), IBD-related QoL (SIBQ), perceived control over IBD (IBD-control), disease activity (HBI, SCCAI, laboratory results), repetitive negative thinking (PTQ), mindfulness skills (FFMQ-SF), self-compassion (SCS-SF), positive mental health (MHC-SF, FACIT-F QoL domains) and objective sleep parameters: TST, SE, SOL, WASO, relative and absolute duration of REM, and relative and absolute duration of deep sleep at post-treatment will be analysed using separate Linear Mixed Effect models, with time, group (MBCT + TAU versus TAU) and Group*Time interaction effects added as fixed effects, while controlling for baseline score of the variable of interest and stratification variables (i.e. centre, sex, IBD type). The same parameters as in the primary analysis will be used for these analyses. In addition, we will use regression analyses to investigate whether more mindfulness practice (MAQ) is also associated with better outcomes.

#### Treatment effects at follow-up

In order to investigate consolidation of the treatment effects over time on both the primary outcome (psychological distress) and secondary outcomes (all secondary outcomes except objective sleep parameters) we will again perform linear mixed effect models with time (T1, T2, T3, T4), group (MBCT + TAU versus TAU) and Group*Time interaction, including the same parameters as mentioned above and controlling for baseline levels and stratification variables. Restricted maximum likelihood will be used as estimation method to handle missing data. Cohen’s *d* will be calculated by dividing the adjusted group difference between the means at T4 by the pooled standard deviation at T0.

We also aim to examine the effect of MBCT on recurrence of disease activity (flares). Therefore, we will perform a proportional hazards model (i.e. Cox regression analysis) to analyse the differences between MBCT + TAU and TAU alone in time to flare within the 12-months study period.

#### Moderation analyses

Moderation analyses will be performed by adding the interaction terms of potential moderators with group to above mentioned post-treatment and follow-up models. We will run these analyses for the effect of group on psychological distress (HADS) and positive mental health (MHC-SF) scores at post-treatment and their course during 12 months follow-up. Separate models will be run for each possible moderator and for both outcomes. The following moderators will be used: age, sex, level of education, IBD type, disease duration, type of IBD medication (aminosalicylates, immunosuppressants, biologicals (including small molecule therapy)), repetitive thinking, mindfulness, self-compassion, psychological distress (HADS) and positive mental health (MHC-SF).

#### Mediation analyses

In order to assess whether the measured effect on psychological distress at posttreatment can be (partially) explained by changes in mindfulness skills, self-compassion, repetitive negative thinking and sleep quality, we will perform mediation analysis following the recommendations by Preachers and Hayes [74] for multiple mediation models. Psychological distress scores at post-treatment will be controlled for its baseline levels in all mediation analyses. Standardised residualised change scores for all potential mediators will be calculated [75]. We will first estimate the indirect effect of the potential mediators using a univariate model. Next, the variables shown to have a mediating role in the univariate models will be entered into a multivariate model to assess their relative contributions. A nonparametric bootstrapping method will be used to assess the indirect effect based on 5000 bootstrapped samples using bias corrected and accelerated 95% confidence intervals as provided by Hayes [76] in the SPSS PROCESS macro version 4.1.

#### Cost-effectiveness

The cost-effectiveness evaluation will be carried out from both a healthcare and societal perspective considering direct as well as indirect costs. Data on resource use (healthcare uptake) and productivity losses will be collected with the TiC-P. Total costs, measured with the TiC-P will be estimated using an incremental bottom-up micro-costing approach, where costs for each patient will be obtained by multiplying information on each element of service with standard costs, based on the Dutch guideline for costing research [67]. We will consider three types of costs: (1) intervention costs of offering MBCT; (2) costs related to healthcare uptake; and (3) costs stemming from productivity losses due to absenteeism. The recall period will be four weeks at baseline, and will consist of three months (the entire period between each follow-up assessment) at 3, 6, 9 and 12 months follow-up. Health related QoL data (based on EQ-5D) from baseline up till 12 months will be used to calculate QALYs using the trapezium rule. Overall mean and median costs and health related QoL will be compared across the conditions and where relevant, differences will be calculated inclusive of 95% confidence intervals. Incremental cost-effectiveness will be determined in terms of incremental costs per QALY gained. The Incremental cost-effectiveness ratio will be determined on the basis of incremental costs and effects of MBCT compared to TAU. Bootstrap simulations with 1.000 replications will be used to get uncertainty intervals in our economic evaluation, taking into account the correlations between costs and QALYs. In addition, a cost-effectiveness acceptability curve will be produced to show the probabilities that given a certain value for the cost-effectiveness threshold, either of the tested strategies is the cost-effective option. In case of missing data, we will use multiple imputation methods to estimate the missing values.

## Discussion

This study will provide more insight in the clinical and cost-effectiveness of MBCT in patients with IBD. It will estimate the effect of MBCT on many different aspects: primarily on psychological distress, but also on sleep quality, fatigue, disease activity, perceived disease control, QoL and positive mental health. Adding objective measures for sleep quality is innovative and important, especially given the moderate correlation between subjective sleep quality measures and polysomnographic measures [37].

If effective, MBCT can be a valuable addition to the available psychosocial interventions for patients with IBD, especially for those with elevated levels of psychological distress. In addition, the embedded process evaluation will hopefully contribute to the implementation of MBCT into clinical practice if it appears to be cost-effective.

Furthermore, the potential health benefits of MBCT and other MBIs may not be restricted to patients with IBD. In line with the current rationale, MBIs may also have a positive effect on psychological well-being, coping and quality of life in patients with other chronic conditions, such as rheumatoid arthritis and multiple sclerosis [77]. Possible mechanisms of effectiveness can serve as a model for improving MBIs for a broader population of people with chronic illness.

## Trial status

This paper complies with version 5 of the research protocol (amendment 4) as approved by the medical-ethical committee (#2021–7319; NL75762.091.20), dated September 6, 2021.

Recruitment started in July 2021 and was completed in September 2022. Last follow-up measurements will be in September 2023.

## Electronic supplementary material

Below is the link to the electronic supplementary material.

Supplementary Material 1

## Data Availability

The datasets used and/or analysed during the MindIBD study will become available from corresponding author on reasonable request after the trial is completed. Results will be disseminated through relevant peer-reviewed journals and scientific conferences. Results will also be shared with participants, mindfulness trainers, healthcare professionals of participating centres and patient association Crohn & Colitis NL through newsletters and presentations.

## List of abbreviations

EEG: Electroencephalography
EQ-5D: EuroQol-5D-5 L
FACIT-F Functional: Assessment of Chronic Illness Therapy-Fatigue
FFMQ-SF: Five Facet Mindfulness Questionnaire-Short Form
HADS: Hospital Anxiety and Depression Scale
HBI: Harvey-Bradshaw Index
IBD: Inflammatory Bowel Disease(s)
ISI: Insomnia Severity Index
MAQ: Mindfulness Adherence Questionnaire
MBCT: Mindfulness-Based Cognitive Therapy
MBI: Mindfulness-based intervention
MBI:TAC: Mindfulness-Based Interventions: Teaching Assessment Criteria
MBSR: Mindfulness-Based Stress Reduction
MHC-SF: Mental Health Continuum-Short Form
PSQI: Pittsburgh Sleep Quality Inventory
PTQ: Perseverative Thinking Questionnaire
QALY: Quality-Adjusted Life Year
QoL: Quality of Life
RCT: Randomised Controlled Trial
REM: Rapid Eye Movement
(S)AE: (Serious) Adverse Event
SCCAI: Simple Clinical Colitis Activity Index
SCS-SF: Self-Compassion Scale–Short Form
SE: Sleep Efficiency
SIBDQ: Short Inflammatory Bowel Disease Questionnaire
SOL: Sleep Onset Latency
TAU: Treatment As Usual
TIB: Time In Bed
TiC-P: Trimbos and iMTA questionnaire for Costs associated with Psychiatric illness
TST: Total Sleep Time
WASO: Wake After Sleep Onset
