# Supplementary material for: Effectiveness of Mindfulness-Based Cognitive Therapy in reducing psychological distress and improving sleep in patients with Inflammatory Bowel Disease: study protocol for a multicentre randomised controlled trial (MindIBD)
Source: BMC Psychol. 2023 Jun 19;11:183. doi: 10.1186/s40359-023-01127-0 (PMC10278305; doi:10.1186/s40359-023-01127-0)
Supplement: Supplementary file 1 — Supplementary Material 1 [file 40359_2023_1127_MOESM1_ESM.docx]

**Appendix 1: informed consent form (translation in English)**

**Belonging to ‘Mindfulness for improvement of psychological distress, fatigue and sleep in patients with IBD’**

- I have read the information sheet. I was able to ask questions. My questions have been answered well enough. I had enough time to decide if I wanted to take part.
- I know that taking part is voluntary. I also know that at any time I can decide not to take part in the study. Or to stop taking part. I do not have to explain why.
- I know that my research data will be used for scientific research and will possibly be published in scientific journals. I agree to this provided that my privacy is guaranteed.
- I know that certain people belonging to the research team can view my data.
- I give the investigator consent to inform my specialist (gastroenterologist) that I am taking part in this study.
- I give the investigator consent to request information from my specialist (gastroenterologist) about faecal calprotectin, haemoglobin, ferritin, C-reactive protein, albumin during the study period.
- I give consent to give my general practitioner or specialist information about accidental discoveries made during the study that are important for my health.
- I give consent to collect and use my data for purposes that are mentioned in this information sheet. The investigators only do this to answer the question of this study.
- I know that some people will be able to see all of my data to review the study. These people are mentioned in this information sheet. I give consent to let them see my data for this review.
- I give permission to the investigators to download sleep data from my Fitbit account. This anonymized data may also be used by third parties.
- I give permission to store my research data for 15 years after the end of this research.
- Please tick yes or no in the table below.

| I give consent to store my data to use for other research, as stated in the information sheet. | Yes ☐ | No☐ |
| --- | --- | --- |
| I give consent to ask me after this study if I want to participate in a follow-up study. | Yes ☐ | No☐ |

- I want to take part in this study.

My name is (subject): ………………………………..

Signature: ……………………… Date : __ / __ / __

-----------------------------------------------------------------------------------------------------------------

I declare that I have fully informed this subject about the study mentioned.

If any information becomes known during the study that could influence the subject's consent, I will let this subject know in good time.

Investigator name (or their representative):……………………………….

Signature:……………………… Date: __ / __ / __

-----------------------------------------------------------------------------------------------------------------

*The study subject will receive a complete information sheet, together with a signed version of the consent form.*
